# A Case of In-Bore Transperineal MRI-Guided Prostate Biopsy of a Patient with Ileal Pouch-Anal Anastomosis

**DOI:** 10.1155/2015/676930

**Published:** 2015-12-30

**Authors:** Michael Kongnyuy, Thomas Frye, Arvin K. George, Amichai Kilchevsky, Amogh Iyer, Meet Kadakia, Akhil Muthigi, Baris Turkbey, Brad J. Wood, Peter A. Pinto

**Affiliations:** ^1^Urologic Oncology Branch, National Cancer Institute, National Institute of Health, Bethesda, MD 20892, USA; ^2^Molecular Imaging Program, National Cancer Institute, National Institute of Health, Bethesda, MD 20892, USA; ^3^Center for Interventional Oncology, National Cancer Institute, National Institute of Health, Bethesda, MD 20892, USA

## Abstract

Ulcerative colitis (UC) is an inflammatory disease that specifically affects the colon. Ulcerative colitis is primarily treated medically and refractory disease is treated with proctocolectomy and ileal pouch-anal anastomosis (IPAA). Gastroenterologists advise against digital rectal exams, pelvic radiation therapy, and transrectal ultrasound (TRUS) biopsies of the prostates of ileal pouch-anal anastomosis patients. Any form of pouch manipulation can lead to severe bleeding, inflammation, and pain. Urologists are therefore faced with the challenge of doing a prostate biopsy without a transrectal ultrasound. We report the rare case of a patient with an ileal pouch-anal anastomosis who underwent in-bore transperineal MRI-guided biopsy of the prostate.

## 1. Introduction

Ulcerative colitis (UC) is characterized by diffuse inflammation of the colonic mucosa. UC, 95% of the time, affects the rectum but never involves the small bowel [1–3]. UC has a prevalence of 500,000 and an incidence of 8–12 per 100,000 population per year in the United States [1]. First line therapy is medical and depends on the location and severity of disease. Medical therapy includes mesalamine, steroids, 5-aminosalicylate, 6-mercaptopurine, azathioprine, infliximab, and thiopurines. Patients with severe colitis refractory to maximal medical therapy undergo either a proctocolectomy with ileostomy, catheterizable stoma, or ileal pouch-anal anastomosis (IPAA) [2, 3].

Ileal pouch-anal anastomosis was first described in the early 1980s and offers bowel continuity with long-term pouch survival of >90% at 20 years of age [3]. Men with IPAAs pose a diagnostic challenge to urologists in regard to prostate cancer detection. Performing a digital rectal exam (DRE) and imaging the prostate of a patient with an IPAA with transrectal ultrasound (TRUS) increase the risk of bleeding and intestinal inflammation [4].

We report the rare case of a patient with an IPAA who underwent in-bore transperineal MRI-guided biopsy of the prostate.

## 2. Case Report

**Figure 1:**
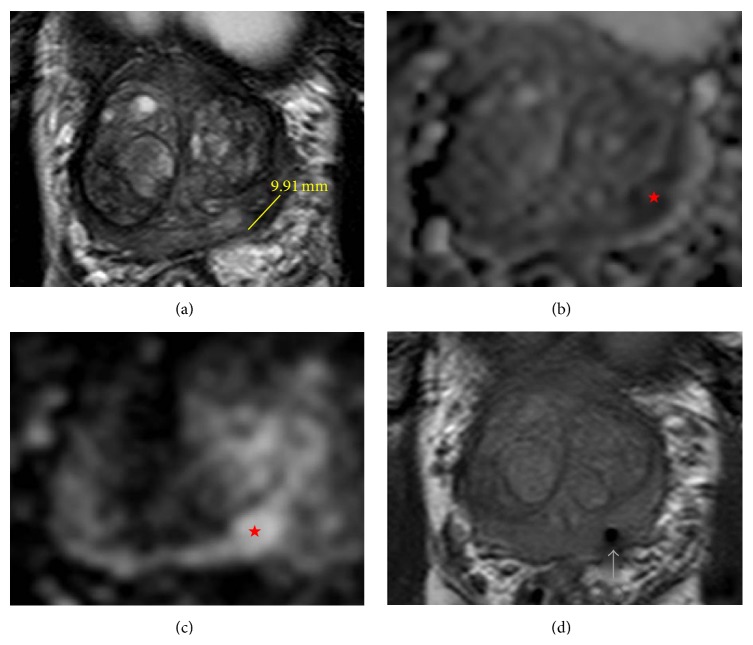
(a) Magnetic resonance T2 weighted axial image of the prostate tumor (left base of the peripheral zone) with line indicating a tumor diameter of 9.19 mm. (b) Magnetic resonance diffusion weighted with apparent diffusion (ADC) mapping; lesion indicated by red star. (c) Magnetic resonance diffusion weighted with B2000 axial image of the prostate tumor (left base of the peripheral zone) indicated by the red star. (d) Magnetic resonance T2 weighted axial image confirming accurate needle placement in lesion prior to taking biopsy (arrow pointing to needle tract).

This is a 75-year-old man with a history of severe refractory UC and benign prostatic hypertrophy who was referred to us because of elevated PSA. His primary care physician performed routine PSA screening and his levels began to increase from 0.77 ng/mL 7 years ago to 3.6 ng/mL (7.2 ng/mL when corrected for finasteride use) most recently. A multiparametric MRI (MP-MRI) of the prostate was performed consisting of T2 weighted and diffusion weighted with apparent diffusion coefficient (ADC) and B2000 mapping images using a 3 Tesla MRI with a 32-channel phased array surface coil (Figures 1(a), 1(b), and 1(c)). The prostate was mildly enlarged (33 cc) secondary to BPH and the peripheral zone had patchy signal intensity. A 9.19 mm lesion (PIRADS 4) with no extracapsular extension (ECE) was identified in the left base of the peripheral zone.

He was diagnosed with UC in his mid-20s and ultimately underwent total proctocolectomy with IPAA 17 years ago. The patient has no other major medical comorbidities. He takes finasteride 5 mg PO daily for BPH and has no family history of prostate cancer. The patient's gastroenterologist recommended against DREs or a TRUS biopsy given the patient's surgical history. This posed a diagnostic dilemma as he likely had a clinically significant prostate cancer given the MRI findings; however, standard TRUS biopsy could not be performed. We decided to proceed with in-bore MRI transperineal targeted biopsy of the suspicious lesion in collaboration with the radiology department.

In the MRI suite, the patient was placed in the supine position and given sedation. A Visualase (Medtronic Inc.) transperineal grid with fiducial markers was placed against the patients' perineum. The left base peripheral zone lesion was targeted using the Visualase software and 4 transperineal biopsy cores were obtained. Once the needle was placed, imaging was performed to confirm accurate needle placement (Figure 1(d)). The patient tolerated the procedure well with no complications. Pathology confirmed a Gleason 4 + 4 = 8 adenocarcinoma of the prostate in one of the 4 cores obtained.

## 3. Discussion

Ulcerative colitis is a type of inflammatory bowel disease that is primarily managed medically; however, about 30% of the cases eventually undergo colectomy for refractory disease [1–3]. The total proctocolectomy with IPAA is the most common surgical treatment [2–4].

The number of UC patients who undergo surgery with IPAA is high and urologists are faced with the challenge of how to screen and properly diagnose these patients. About 30% of UC patients eventually undergo colectomy for medically refractory disease [4]. The increased risk of bleeding due to small bowel hypervascularity and anastomotic leaks from ultrasound trauma are basis for the GI recommendation of avoiding DREs, TRUS, or any form of radiation treatment [3]. With a rising PSA, standard protocol is a 12-core systematic TRUS biopsy [5], which is not an option for our patient because of his IPAA. Advances in prostate MP-MRI in characterizing suspicious lesions and the advent of software platforms like Visualase [6, 7] allowed us our first ever in-bore MRI-guided transperineal prostate biopsy with no TRUS of a UC patient with IPAA.

This was collaborative effort between urology and radiology. It is important to note that the highest yield for cancer detection is from the first biopsy pass as the prostate can move and rotate slightly resulting in inaccurate subsequent biopsies. The final pathology showed high-risk disease, Gleason 4 + 4 = 8, and highlights the importance of the urologist to have and be familiar with the different diagnostic techniques when faced with unique patient populations.

Colorectal cancer involving the anal sphincter and refractory cases of UC (not amenable for IPAA) are routinely treated with abdominoperineal resection with anal closure [8]. Though relatively infrequent, these patients may develop subsequent suspicion of prostate cancer requiring biopsy to provide a histologic diagnosis. Shinohara et al. [9] were able to visualize the prostate by placing the TRUS probe onto the surface of the skin of the perineum. The quality of the images they acquired was compromised and did not provide distinct visualization of the capsule or internal anatomy of the prostate. This can increase the difficulty to visualize the needle or biopsy within the same plane. In-bore MR targeting provides the additional benefit of improved cancer detection, which must be balanced, with the added cost of personnel, MRI time, and equipment in this narrow subset of patients.

## Abbreviations

TRUS: Transrectal ultrasound
IPAA: Ileal pouch-anal anastomosis
UC: Ulcerative colitis
MR: Magnetic resonance
MRI: Magnetic resonance image
MP-MRI: Multiparametric magnetic resonance imaging
DRE: Digital rectal exam
PSA: Prostate specific antigen
ECE: Extra capsular extension
BPH: Benign prostatic hypertrophy
GI: Gastrointestinal
PO: Per os
PIRADS: Prostate Imaging Reporting and Data System.
